# *Quinquelaophonte enormis* sp. nov., a new interstitial copepod (Harpacticoida: Laophontidae) from Korea

**DOI:** 10.7717/peerj.10007

**Published:** 2020-09-22

**Authors:** Jaehyun Kim, Eunjung Nam, Wonchoel Lee

**Affiliations:** 1 Department of Life Science, Hanyang University, Seoul, South Korea; 2 Animal Resources Division, National Institute of Biological Resources, Incheon, South Korea

**Keywords:** Meiofauna, DNA barcoding, Sandy habitat, East sea

## Abstract

We collected an undescribed laophontid copepod from a coarse sand habitat on the east coast of Korea and named it *Quinquelaophonte enormis* **sp. nov**. We compared the detailed morphological characteristics of the new species with those of congeneric species. Among them, the new species shows a superficial resemblance to the Californian species *Quinquelaophonte longifurcata* Lang, 1965. However, the two species are easily distinguishable by the setation of the syncoxa on the maxilliped and the fourth swimming leg. The new species has the variable setation on the second to fourth swimming legs. The variations appear among individuals or between the left and right rami of a pair of legs in a single specimen. Although complex chaetotaxical polymorphism occur in this new species, we used myCOI and Cytb to confirm that the new species is not a species complex. Also, partial sequences of 18S and 28S ribosomal RNA genes were used to analyze the position of the new species within the family Laophontidae. The new species****is the fourteenth *Quinquelaophonte* species in the world and the second species in Korea.

## Introduction

The harpacticoid copepod, Laophontidae Scott T., 1904 is one of the largest families consisting of more than 320 valid species in 75 genera (Ahyong et al., 2011; Huys & Lee, 2018). Considering the large number of members, DNA information about this family is lacking. Although the available sequences are few, Yeom et al. (2018) analyzed the phylogenetic relationships among five laophontid genera (*Laophontina* Norman & Scott T., 1905; *Microchelonia* Brady, 1918; *Paralaophonte* Lang, 1948; *Pseudonychocamptus* Lang, 1944; *Vostoklaophonte* Yeom et al., 2018 using partial sequences of 18S ribosomal RNA (rRNA) gene and revealed a close relationship between *Vostoklaophonte* and *Microchelonia*. In addition, Wells (2007) noted that this family has variable setation on the second to fourth swimming legs. The variation can appear within a population or between populations, between the sexes and, even, between the left and right rami of a pair of legs in a single individual.

The genus *Quinquelaophonte* Wells, Hicks & Coull, 1982 was raised to accommodate species incorporated in the *quinquespinosa*-group of *Heterolaophonte* Lang, 1948 with the description of a new species, *Q. candelabrum*, collected from New Zealand. Also, they queried the description of *Laophonte brevicornis* (Scott T., 1894) regarding the presence of an inner seta on the first exopodal segment of the fourth swimming leg. Although this condition was unknown in any other laophontid copepods and no males had been found, Wells, Hicks & Coull (1982) confirmed it as a species *incertae sedis* near or perhaps within the genus *Quinquelaophonte* based on several morphological features. Since the genus was erected, 13 species have been reported from the brackish and marine benthic habitats of around the world, including the puzzling species, *Q. brevicornis*. Afterward, Lee (2003) suggested that *Q. quinquespinosa* was a complex of several distint species and described *Q. koreana* which was the sole *Quinquelaophonte* species in Korea. This species was known to have an inner seta on the second exopodal segment of the fourth swimming leg like most of *Quinquelaophonte* species, but its caudal rami were described as shorter than all congeners. *Quinquelaophonte aurantius* was reported by Charry et al. (2019) as the second species of New Zealand following *Q. candelabrum*. When *Q. aurantius* was described, they referred to *Q. parasigmoides* and *Q. wellsi* known from Réunion Isle (east of Madagascar) and South Australia, respectively, as the most similar species based on the morphological comparison, not *Q. candelabrum* with the similar distribution. Furthermore, the partial sequences of mitochondrial cytochrome oxidase I (mtCOI) and 18S rRNA gene of *Q. aurantius* were given as the first genetic information published for *Quinquelaophonte*. Of these, using 18S rDNA phylogeny, they revealed that the genus *Quinquelaophonte* has a close relationship with two genera, *Laophontina* and *Pseudonychocamptus* more than *Microchelonia*, *Paralaophonte* and *Vostoklaophonte*. Within this genus, some species are known to have variable setation as Wells (2007) mentioned (Table 1).

**Table 1:** Comparison of morphological features of swimming legs of *Quinquelaophonte* species except for *Q. brevicornis* (Scott T., 1894).

Species	References	P1		Setal formulae ♀(♂)					
		Types of elements on exp-2	Accessory seta on enp-2	P2		P3		P4	
				exp	enp	exp	enp	exp	enp
*Quinquelaophonte enormis***sp. nov.**	A	2 setae + 3 spines	short	0. 1.123 (0.1.123)	0.120 (0.0–120)	0.1.0-123 (0.1.0–123)	0.221 (0.221)	0.0 − 1.022 − 3 (0.0–1.023)	0.120 (0.120)
*Q. aestuarii*	B	2 setae + 3 spines	short	0.1.123 (0.1.123)	0.120 (0.120)	0.1.123 (0.1.123)	0.221 (0.221)	0.1.123 (0.1.123)	0.120 (0.120)
*Q. aurantius*	C	2 setae + 3 spines	short	0.1.123 (0.1.123)	0.120 (0.120)	0.1.123 (0.1.0–123)	0.221 (0.221)	0.1.123 (0.0–1.0–123)	0.120 (0.120)
*Q. bunakenensis*	D	2 setae + 3 spines	short	0.1.123 (0.1.123)	0.120 (0.120)	0.1.223 (0.0–1.222–3)	0.220 (0.220)	0.1.123 (0.1.123)	0.120 (0.120)
*Q. candelabrum*	E	2 setae + 3 spines	short	0.1.123 (0.1.213)	0.120 (0.120)	0.1.223 (0.1.223)	0.221 (0.221)	0.1.123 (0.1.222)	0.120–1 (0.121)
*Q. capillata*	F	2 setae + 3 spines	–	(0.1.123)	(0.120)	(0.1.123)	(0.222)	(0.1.123)	(0.220)
G	5 setae	–	0.1.123 (0.1.123)	0.120 (0.120)	0.1.123 (0.1.222)	0.221 (0.221)	0.1.123 (0.1.123)	0.111 (0.120)
H	2 setae + 3 spines	short	0.1.123 (0.1.123)	0.120 (0.120)	0.1.123 (0.1.123)	0.221 (0.221)	0.1.123 (0.1.023)	0.120 (0.120)
*Q. koreana*	I	2 setae + 3 spines	short	0.1.123 (0.1.123)	0.120 (0.120)	0.1.223 (0.1.223)	0.221 (0.221)	0.1.123 (0.1.123)	0.121 (0.121)
*Q. longifurcata*	J	2 setae + 3 spines	short	0.1.123 (0.1.123)	0.120 (0.120)	0.1.0-123 (0.1.123)	0.221 (0.221)	0.1.023 (0.0.023)	0.121 (0.121)
*Q. parasigmoides*	K	2 setae + 3 spines	short	0.1.123 (0.1.123)	0.120 (0.120)	0.1.123 (0.1.1–223)	1.321 (0–1.2–321)	0.1.123^a^(0.1.123)	0.120 (0.120)
*Q. prolixasetae*	L	5 long setae	long	0.1.123^b^(0.1.123)	0.120 (0.120)	0.1.123 (0.1.123)	0.221 (0.221)	0.1.023 (0.1.023)	0.120 (0.120)
*Q. quinquespinosa*	M	2 setae + 3 spines	short	0.1.123 (0.1.123)	0.120 (0.120)	0.1.223 (0.1.223)	0.221 (0.220)	–	0.120
	N	2 setae + 3 spines	short	0.0.123 (0.1.123)	0.120 (0.120)	0.1.223 (0.1.223)	0.221 (0.221)	0.1.123 (0.1.123)	0.121 (0.121)
H	2 setae + 3 spines	short	0.1.123	0.120	0.1.223	0.221	0.1.223	0.11-21
*Q. varians*	O	2 setae + 3 spines	short	0.1.123 (0.1.123)	0.120 (0.120)	0.1.123 (0.0.213)	0.221 (0.121)	0.1.123 (0.0.212–3)	0.120 (0.120)
*Q. wellsi*	P	2 setae + 3 spines	long	0.1.123 (0.1.123–4)	0.120 (0.120)	0.1.223 (0.1.1–223)	0.221 (0.221)	0.0-1.123 (0.1.123)	0.1–220 (0.120)

**Notes.**

A this study B Sciberras, Bulnes & Cazzaniga, 2014 C Charry et al., 2019 D Mielke, 1997 E Wells, Hicks & Coull, 1982 F Wilson, 1932 G Coull, 1976; Coull, 1986 H Gómez & Morales-Serna, 2013 I Lee, 2003 J Lang, 1965 K Bozic, 1964; Bozic, 1969 L Walker-Smith, 2004 M Sewell, 1924 N Wells & McKenzie, 1973 O Bjornberg, 2010 P Hamond, 1973

a Bozic (1969) mentioned morphological differences between materials of La Réunion and Bermudes. The former was described to have six elements on the female P4 exp-3, and the latter had seven. However, Bozic (1969) did not explain where an element in the latter was increased.

b Walker-Smith (2004) noted that the armature formula of P2 exp-3 was “123”, but 5 elements were presented in the female P2 exp-3 (Fig. 3C in Walker-Smith, 2004).

In taxonomic studies, the chaetotaxy of various appendages has been used for many crustaceans such as amphipods, isopods, ostracods, tanaidaceans, and copepods (Wells, 2007; Brad et al., 2015; Kim, Lee & Karanovic, 2017; García-Herrero, Sánchez & García-Gómez, 2019; Yoo et al., 2019). In taxonomy of copepods in particular, the arrangement, size, and type of setae and spines on the swimming legs are used as major keys for species identification (Huys et al., 1996; Chihara & Murano, 1997; Wells, 2007). However, some infrafamilial taxa of Harpacticoida are known to have intraspecific variable setation that differ in number (Lang, 1965; Wells, 2007; George, 2008; Gheerardyn & Veit-Köhler, 2009; Bang, Lee & Lee, 2011; Menzel, 2011). In some cases, a single species thought to have variable setation within and between populations has in fact been shown to be several distinct species through morphological and genetic analyses (Fleeger, 1980; Rocha-Olivares, Fleeger & Foltz, 2001; Gómez et al., 2004; Garlitska et al., 2012; Fiers & Kotwicki, 2013).

During a sampling of meiofaunal organisms inhabiting sandy sediment on the east coast of Korea, an undescribed species of Laophontidae showing chaetotaxical polymorphism was collected. In this study, those animals were assigned to a new *Quinquelaophonte* species, and descriptions and illustrations are provided. Additionally, phylogenetic trees were generated using partial sequences of 18S and 28S rRNA genes to analyze the position of the new species within the family Laophontidae. Partial information for two mitochondrial genes, mtCOI and cytochrome b (Cytb), are also given as DNA barcodes herein.

## Materials & Methods

**Sample collection.** The specimens of the new species were collected from a small sandy shore in Busan, Korea (35°16′3.95″N; 129°14′39.72″E), on 13 April 2017 using the Karaman-Chappuis sampling method. Samples were filtered with a 63 µm mesh sieve and washed several times with fresh water. The residuary material was then preserved in 94% ethanol. Isolation of specimens was carried out in a laboratory under a SZX-7 stereo microscope (Olympus, Japan). The sorted specimens were transferred to 99% ethanol and stored at −20 °C.

**DNA extraction.** Prior to extraction, specimens were washed with distilled water for about 30 min to remove ethanol. The total genomic DNA (gDNA) was extracted from each whole specimen without homogenization using 200 µl of 10% solution of Chelex^®^ 100 resin (50–100 mesh, sodium form; Sigma-Aldrich, USA), and then it was used as a DNA template without purification. After gDNA isolation, the remaining exoskeleton of each specimen was carefully transferred to distilled water for morphological analyses such as observation, line drawing, and taking photographs with scanning electron microscope (SEM).

**Observation.** We prepared 13 gDNA extracted and non-extracted specimens on slides in lactophenol and observed them under a BX-51 compound microscope (Olympus, Japan). Some specimens were dissected using a tungsten needle in lactophenol or lactic acid for more detailed observation. All dissections were made using a SZX-7 stereo microscope (Olympus, Japan). Pencil drawings of the whole body and the dissected appendages were made with a drawing attachment on a BX-51 compound microscope. Using iPad Pro and Apple Pencil (Apple, USA), line drawings were conducted in the PROCREATE application (Savage Interactive, Australia). All specimens observed in this study were mounted on slides in lactophenol and sealed with transparent nail varnish.

The exoskeletons remaining after extracting the gDNA of one female and four males were used for in-depth observation through SEM. The specimens were dehydrated in 99% ethanol for about 30 min without a graded ethanol series. For sample drying, hexamethyldisilazane (HMDS, (CH_3_)_3_SiNHSi(CH_3_) _3_) was used (Braet, De Zanger & Wisse, 1997; Shively & Miller, 2009). Each dehydrated specimen was submerged in HMDS in a six-well plate, and the plate was placed in a 50 °C oven until the HMDS had evaporated completely. The dried specimens were directly attached to SEM stubs using conductive double-sided carbon tape or stuck on the tip of a fine metallic pin such as tungsten wire or a staple with nail varnish or carbon tape to enable observation of the various sides of each specimen. Prepared SEM stubs were coated with gold in an ion-coater, SPT-20 (Coxem, Korea). In an EM-30 SEM (Coxem, Korea), specimens coated on stubs were examined and photographed.

All materials prepared in this study were deposited in the National Institute of Biological Resources (Incheon, Korea).

**DNA amplification and genetic analysis.** Two nuclear rRNA, 18S and 28S, and two mitochondrial, mtCOI and Cytb, genes were partly amplified through polymerase chain reaction (PCR) using an AccuPower^®^ PCR premix (Bioneer, Korea) in TaKaRa PCR Thermal Cycler Dice (Takara Bio, Japan). Each premix tube was prepared with 2 µl of the genomic DNA template, 1 µl of forward and reverse primers, and 16 µl of distilled water. The pairs of primers used to amplify each locus are as follows: 18S rDNA, 18A1 mod and 1800 mod (Raupach et al., 2009) at annealing temperature of 50 °C for 36 cycles; 28S rDNA, 28S-F1a and 28S-R1a (Ortman, 2008) at 51 °C for 36 cycles; mtCOI, LCO-1490F (Folmer et al., 1994) and COP-COI-2189R (Bucklin et al., 2010) at 45 °C for 40 cycles; Cytb, UCYTB151F and UCYTB270R (Merritt et al., 1998) at 42 °C for 40 cycles. All PCR products were run on a 1% Tris acetate-EDTA agarose gel for 40 min at a voltage of 70V with a 100 bp DNA ladder (Bioneer, Korea). The PCR amplicons were purified through ethanol precipitation in advance of sequencing. The purified PCR products were sequenced on an ABI automatic capillary sequencer (Macrogen, Korea) with the same primer sets used for amplification. Four additional primers, F1, CF2, CR1, and R2 (Laakmann et al., 2013), were employed for 18S rRNA sequencing.

The forward and reverse strand chromatograms obtained in this study were visualized, trimmed, edited and assembled using Geneious Prime 2020.1 (https://www.geneious.com). The assembled nucleotide sequences were identified using BLAST (Altschul et al., 1990).

To check whether our materials were a complex of distinct species, two mitochondrial sequences were used to measure the pairwise distance. The uncorrected pairwise distance (*p*-distance) was calculated with Mega X v10.1.7 (Stecher, Tamura & Kumar, 2020) because the use of K2P distance for DNA barcoding analyses is under debate (Collins & Cruickshank, 2013). The mtCOI sequence for *Q. aurantius* (MH444814) was obtained for the calculation.

Among the obtained sequences, two nuclear rRNA genes were used for the phylogenetic analysis to investigate the placement of the new species within Laophontidae. The molecular data from the two rRNA genes were aligned, separately, together with the sequences of other harpacticoids downloaded from GenBank (Tables S1 –S2), using MAFFT v7.313 (Katoh & Standley, 2013). Subsequently, the aligned sequences were curtailed at 1918 base pairs (bp) for 18S and 873 bp for 28S. *Canuella perplexa* (18S: EU370432; 28S: MF109111) was selected as the outgroup as in the previous studies (Yeom et al., 2018; Charry et al., 2019). We analyzed each gene independently using Bayesian methods. The best-fitting evolutionary models were computed in jModeltest v2.1.10 (Darriba et al., 2012)*.* The chosen models were TIM1+I+G for 18S and TrN+I+G for 28S. Bayesian analyses were conducted using in MrBayes 3.2.7a (Ronquist et al., 2012). Because the selected models are not implemented in MrBayes, substitution models were replaced by GTR+I+G. Two independent analyses were carried out for each gene in two parallel runs with four chains and they stopped automatically when the average standard deviation of split frequencies was below 0.01 (sampling trees every 100 generations, calculating diagnostics every 1,000 generations). The initial 25% of the sampled data was discarded as burn-in, and a majority-rule 50% consensus tree was generated.

**Terminology and abbreviation.** The morphological description follows the terminology proposed by Huys et al. (1996). Abbreviations used in the text are: ae, aesthetasc; enp, endopod; exp, exopod; benp, baseoendopod; enp-1–enp-3, first to third endopodal segments; exp-1–exp-3, first to third exopodal segments; P1–P4, first to fourth swimming legs; P5–P6, fifth to sixth legs.

All type materials were given a deposit number from NIBRIV0000865933 to 0000865949. The meaning of abbreviation is: NIBR, National Institute of Biological Resources; IV: invertebrate.

The electronic version of this article in Portable Document Format (PDF) will represent a published work according to the International Commission on Zoological Nomenclature (ICZN), and hence the new names contained in the electronic version are effectively published under that Code from the electronic edition alone. This published work and the nomenclatural acts it contains have been registered in ZooBank, the online registration system for the ICZN. The ZooBank LSIDs (Life Science Identifiers) can be resolved and the associated information viewed through any standard web browser by appending the LSID to the prefix http://zoobank.org/. The LSID for this publication is: [urn:lsid:zoobank.org:pub:D3FBC69D-D672-459C-A7F3-4ADBC92C5615]. The online version of this work is archived and available from the following digital repositories: PeerJ, PubMed Central and CLOCKSS.

## Results

**Table:** 

Order Harpacticoida Sars G.O., 1903
Family Laophontidae Scott T., 1904
Genus *Quinquelaophonte*Wells, Hicks & Coull, 1982
*Quinquelaophonte enormis***sp. nov.** Kim, Nam & Lee, 2020 (Figs. 1–8)
urn:lsid:zoobank.org:act:7094B4E4-DE75-4C9B-8746-F02EB077B579

**Diagnosis.** Body surface covered with small processes dorsolaterally and all somite without hyaline frills. Caudal ramus about 3.5 times as long as width and with seven setae. Antennule six-segmented in female and eight-segmented in male. Exopod of antenna with three setae. Syncoxa of maxilliped with two setae. P1 exp-2 with two setae and three spines. P1 enp-2 with short accessory seta. P4 exp-3 without inner seta. P4 enp-2 without outer seta. Female P5 with six and five setae on the exopod and beseoendopod, respectively. Male P5 with five setae.

**Type locality.** Sandy shore in Gijang, Busan, east coast of Korea (35°16′3.95″N; 129°14′39.72″E). All specimens were collected from the type locality on 13 April 2017.

**Material examined.** Holotype: 1♀ on a slide (NIBRIV0000865933). Paratypes: Paratype 1, 1♀ dissected on four slides (NIBRIV0000865934); Paratype 2, 1♀ dissected on seven slides (NIBRIV0000865935); Paratype 3, 1♀ dissected on 10 slides (NIBRIV0000865936); Paratype 4, 1♀ dissected on nine slides (NIBRIV0000865937); Paratype 5, 1♂ dissected on eight slides (NIBRIV0000865938); Paratype 6, 1♂dissected on six slides (NIBRIV0000865939); Paratypes 7–12, 6♂ ♂ on each slide (NIBRIV0000865940–0000865945); Paratype 13, 2♂ ♂, *in toto*, on a SEM stub (NIBRIV0000865946); Paratypes 14–16, 1♀2♂ ♂ on each SEM stubs (NIBRIV0000865947–0000865949).

**Figure 1:**
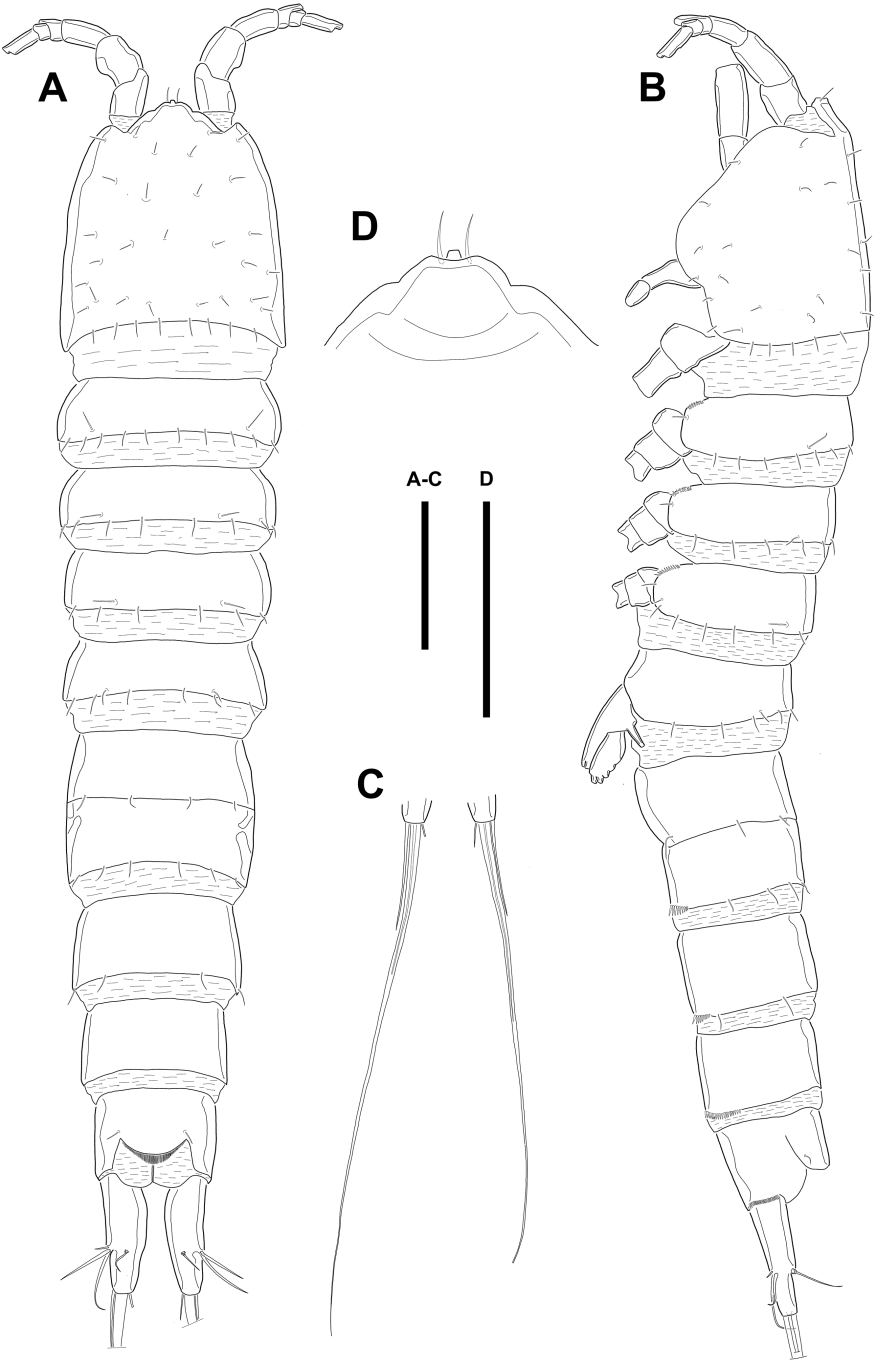
Line drawings of *Quinquelaophonte enormis* sp. nov. Female (holotype). (A): Habitus, dorsal; (B): Habitus, lateral; (C): Terminal part of caudal rami; (D): Roustrum, dorsal. Scale bars: (A–C), 100 µm; (D), 50 µm.

**Description of female.** Body (Fig. 1) length, measured from anterior tip of rostrum to the posterior margin of the caudal rami, 760–916 µm (*n* = 3, mean = 827 µm). All body somite without hyaline frills (Figs. 1A–1B) and entire surface, except for ventral surface, covered with tiny spiniform processes (omitted in line drawings).

Prosome (Figs. 1A–1B, 1D) four-segmented, comprising cephalothorax (cephalosome fused with first pedigerous somite) and three free pedigerous somites. Cephalothorax about as long as three succeeding prosomites combined. Rostrum (Fig. 1D) small, pointed anterior apex, fused to cephalothorax, and with two sensilla near the anterior margin.

**Figure 2:**
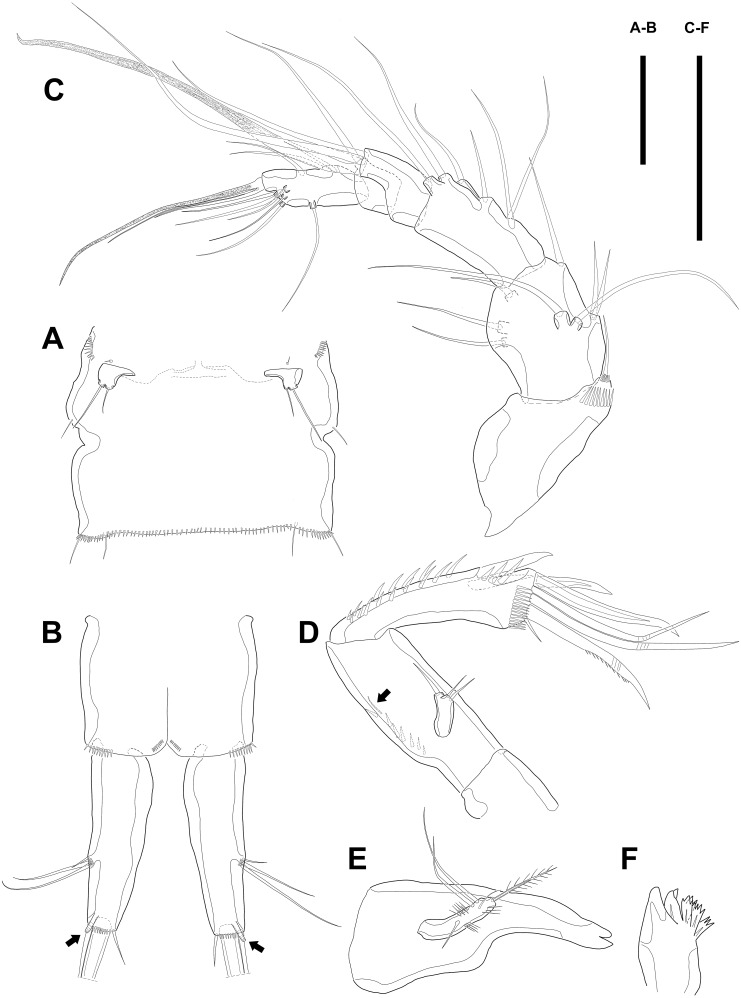
Line drawings of *Quinquelaophonte enormis* sp. nov. Female A: paratype 1; B–E: paratype 2; F: paratype 4). (A) Genital double-somite, ventral; (B) Anal somite and caudal rami (arrows indicate tube pores), ventral; (C) Antennule, ventral; (D) Antenna (arrow indicates an abexopodal seta); (E) Mandible; (F) Gnathobase. Scale bars: (A–B), 100 µm; (C–F), 50 µm.

Urosome (Figs. 1; 2A–2B) gradually tapering posteriorly, five-segmented, and consisting of P5 bearing somite, genital double-somite (second urosomite fused to succeeding somite), two free abdominal somites, anal somite and caudal rami. Genital double-somite with dorsal and lateral ridge marking trace of the original segmentation and completely fused ventrally. Genital field with widely separated pair of P6 and sensilla near P6, anteriorly. P6 with small protuberance bearing two bare setae, of which the outer longer than the inner. Copulatory pore unobserved. Anal somite with sensilla on each side of anal operculum. Caudal rami elongated, tapering posteriorly, about 3.5 times as long as width, with seven naked setae on each ramus: setae I–III arising from the posterior half of the outer margin, seta I shortest, and seta II shorter than seta III; setae IV and V separated and seta V longest; seta VI much shorter than seta V; seta VII located in terminal half of dorsal surface and tri-articulate at base. Spinular row presented on ventral margin, distally, and tube pore (arrowed in Fig. 2B) arising on outer margin ventrally.

Several sensilla presented on all of body somite except for penultimate urosomite, as figured.

Antennule (Fig. 2C) six-segmented, with aesthetasc on each of the fourth and distal segments. First segment slightly protruding on outer margin and armed with two rows of spinules on anterior margin, of which posterior row much longer. Second segment slightly longer than width. Third segment about 1.5 times as long as width and about as long as the preceding segment. Fourth segment with terminal aesthetasc fused basally to one naked seta. Fifth segment shortest. Terminal segment longer than two preceding segments combined and with apical acrothek consisting of aesthetasc fused basally to two naked setae. Armature formula: 1-[1], 2-[8], 3-[6], 4-[1+(ae+1)], 5-[1], 6-[9 + acrothek].

Antenna (Fig. 2D) consisting of coxa, allobasis, free one-segmented endopod and exopod. Coxa small and bare. Allobasis elongated, about 2.5 times longer than width, without distinct surface traces indicating original segmentation and with one small abexopodal seta on median surface (arrowed in Fig. 2D). Endopod as long as allobasis, approximately, and with two stout spines on the distal half of lateral margin. Terminal armature consisting of two bare spines, three geniculate setae, and one tiny seta. Endopod ornamented with two rows of spinules laterally and transverse frills subapically. Exopod arising from one-third of posterior surface of allobasis and with three tiny setae.

Mandible (Figs. 2E–2F) with well-developed gnathobase bearing several multi-dentate teeth on distal margin. Mandibular palp small. Basis with one pinnate seta apically and ornamented with several rows of long spinules. Endopod and exopod fused to basis and armed with three and bare setae, respectively.

**Figure 3:**
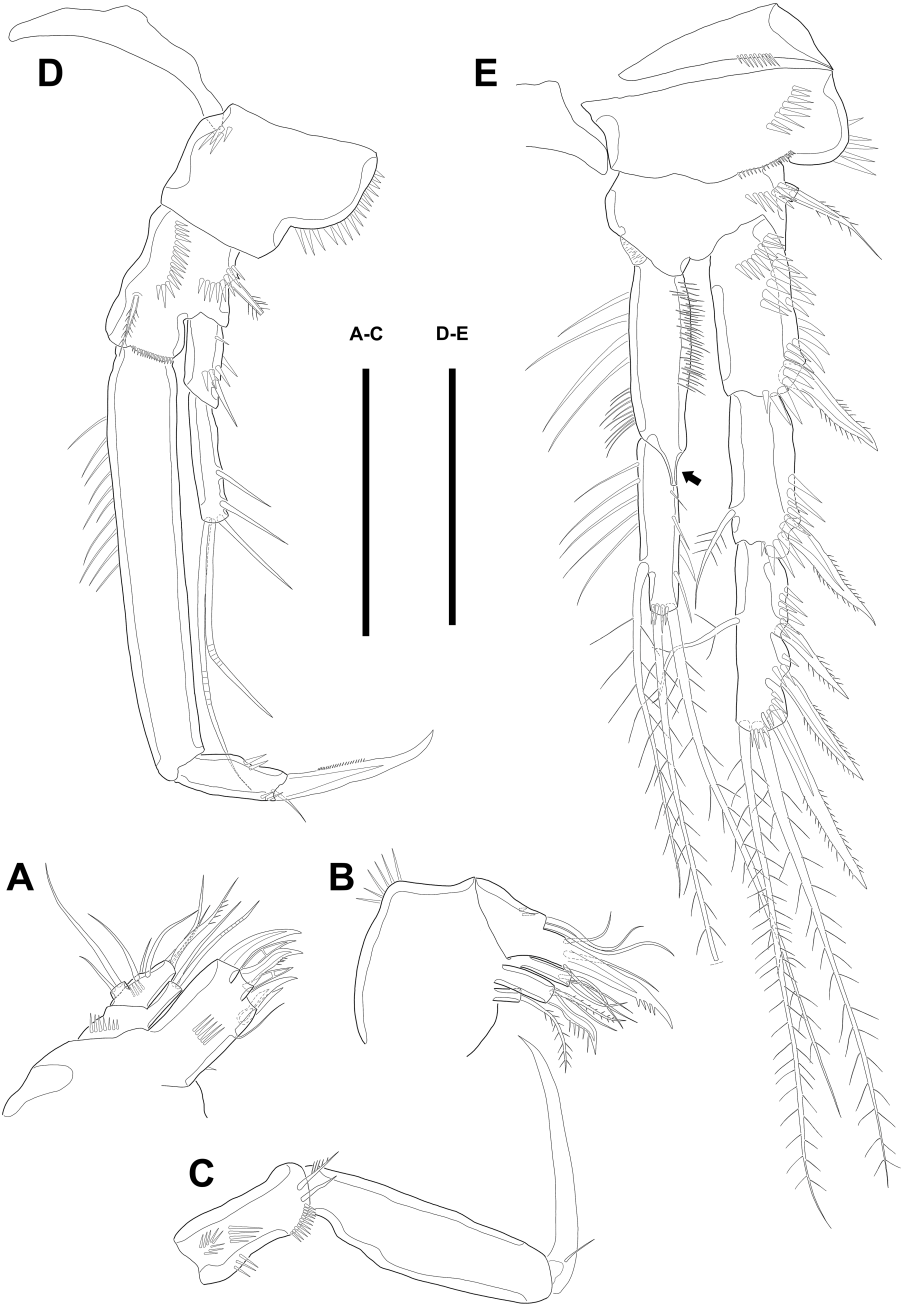
Line drawings of *Quinquelaophonte enormis* sp. nov. Female (A–C, E) paratype 2; (D) paratype 3). (A) Maxillule; (B) Maxilla; (C) Maxilliped. (D) P1; (E): P2. Scale bars: 50 µm.

Maxillule (Fig. 3A). Arthrite strongly developed, with seven spines around the terminal margin, one bare seta on distal half margin, and one row of spinules on posterior surface. Coxa with one endite bearing one bare and one geniculate seta. Basis with one endite bearing one pinnate seta or spine and two bare setae on the terminal margin and one row of spinules on anterior surface. Endopod fused to basis basally and with three naked setae. Exopod small, one-segmented, and with two bare setae.

Maxilla (Fig. 3B) comprising syncoxa, allobasis, and endopod. Syncoxa slightly curved and ornamented with one row of long spinules on distal corner and three cylindrical endites. Praecoxal endite with one pinnate seta and small. Proximal coxal endite with one strong and pinnate spine, one bipinnate seta, and one naked seta. Distal coxal endite with three bare setae. Allobasis elongated, forming to strong, pinnate, and slightly curved spiniform distally and with three bare setae. Endopod represented by two naked setae.

Maxilliped (Fig. 3C) consisting of syncoxa, basis and endopod. Syncoxa about 2 times as long as width and ornamented with one pinnate and one bare seta near terminal margin and several rows of spinules. Basis naked and about 3.5 times as long as width. Endopod elongated, forming to naked claw, and with one tiny bare seta.

**Figure 4:**
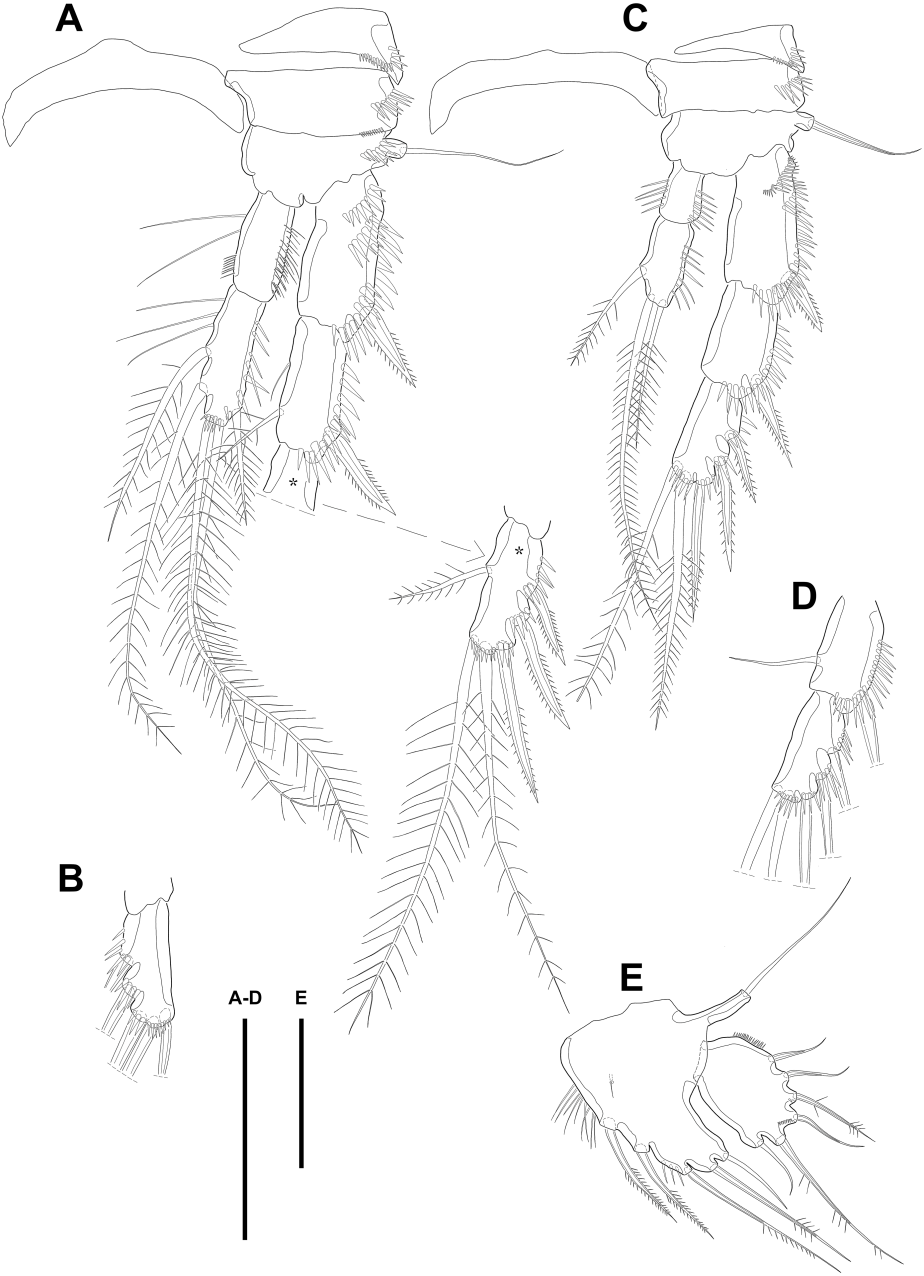
Line drawings of *Quinquelaophonte enormis* sp. nov. Female. (A–C). Paratype 2; (D) paratype 4; (E) paratype 1. (A) Left leg of P3; (B) right distal segment of P3 exp; (C) left leg of P4; (D) left distal segment of P4 exp; (E) P5. Scale bars: 50 µm.

Swimming legs P1–P4 (Figs. 3D–3E; 4A–4D) with wide intercoxal sclerite, three-segmented exopod, and two-segmented endopod (except for P1). P1 exopod two-segmented.

P1 (Fig. 3D). Coxa large, ornamented with one row of spinules on outer margin laterally and one patch of spinules near proximal inner corner of anterior surface. Basis with two bipinnate spines, outer one on half margin and inner on distal half margin and armed with three rows of spinules along anterior outer margin, on anterior margin vertically, and near boundary with endopod, respectively. Exopod about half as long as Enp-1. Exp-1 with one outer spine and one row of spinules from median to outer margin, anteriorly. Exp-2 with three outer spines and two geniculate setae, of which inner one longer than Exopod. Enp-1 elongated and with long setules from proximal half to median of inner margin. Enp-2 with strong claw, tiny seta, and several spinules.

P2 (Fig. 3E). Praecoxa well developed, ornamented with one row of spinules on anterior margin. Coxa with one row of strong spinules laterally, one row of spinules on anterior surface, and one row of short spinules near distal outer corner. Outer margin of basis much narrower than coxa, and with one bipinnate spine and row of spinules. Exopod longer than endopod. Exp-1–3 sub-equal in length and tapering slightly distally. Exp-1 shorter than two succeeding segments combined and with one strong outer bipinnate spine, two rows of spinules from median to outer margin anteriorly, one row of spinules along outer margin, and one row of spinules from near boundary with exp-2 to half of outer margin. Exp-2 with one plumose inner seta and strong bipinnate outer spine and ornamented with one row of spinules along outer margin. Exp-3 with six elements: one plumose inner seta, two apical plumose setae, and three bipinnate spines and four rows of spinules presented near base of the most outer four elements. Endopod reaching to approximately half of exp-3. Enp-1 ornamented with long setules or spinules on the inner and outer margin, as figured, and one tube pore on anterior surface distally (arrowed in Fig. 3E). Enp-2 slightly shorter than preceding segment, ornamented with long setules and spines, as figured, and with three plumose setae, one inner and two apical and one row of spinules on distal outer corner, anteriorly.

P3 (Figs. 4A–4B). Praecoxa well developed, ornamented with one row of spinules around anterior distal corner. Coxa with one row of strong spinules near median outer surface, anteriorly, and one anterior row of short spinules in distal outer corner. Outer margin of basis much shorter than inner and with one bare outer seta arising from setophore and one row of spinules near base of basal seta. Exp-1–3 sub-equally in length and tapering slightly distally. Exp-1–2 as in P2 except for one setule on inner margin of exp-2 laterally. Exp-3 with one plumose inner seta, two plumose terminal setae, and three bipinnate outer spines. Several spinules located near base of setae or spines, except for inner seta. Endopod reaching to end of exp-2, approximately. Enp-1 slightly shorter than enp-2 and armed with several setules on inner and outer margin laterally. Enp-2 with five plumose setae, two on inner, two apical, and one on outer margin. Three long setules and several spinules presented on inner margin laterally and from terminal to outer margin, respectively.

P4 (Figs. 4C–4D)*.* Praecoxa well developed and ornamented with spinular patch around anterior distal corner. Coxa with one row of strong spinules near the median outer surface anteriorly. Outer margin of basis much shorter than inner and with one bare outer seta arising from setophore. Exopod about twice as long as than endopod. Exp-1–3 gradually tapering distally. Exp-1 as in P2–P3, except for longer than each of two following segments. Exp-2 with one strong bipinnate outer spine and armed with spinules from terminal to outer margin. Exp-3 with two plumose apical setae and three bipinnate outer spines. Several spinules presented near base of setae and spines. Endopod reaching to half of exp-2. Enp-1 shorter than enp-2 and ornamented with setules or spinules along inner and outer margins. Enp-2 armed with long setules or spinules on outer margin and with one inner seta and two terminal setae, all of which plumose. Setal formula of P2–P4 as in Table 1.

P5 (Fig. 4E) separated, comprising baseoendopod and exopod. Baseoendopod without distinct surface sutures marking original segmentation, with slender outer setophore bearing one naked seta, and ornamented with long setules on inner margin and sensilla or setule near anterior margin. Endopodal lobe slightly shorter than exopod, with two pinnate inner setae and three apical setae, pinnate, bipinnate, and bare from inner to outer seta, respectively. Exopod sub-ovoid, about 1.5 times as long as width, with six setae, and ornamented with two rows of spinules, as figured. All setae on P5 arising from distinct cylindrical processes.

**Figure 5:**
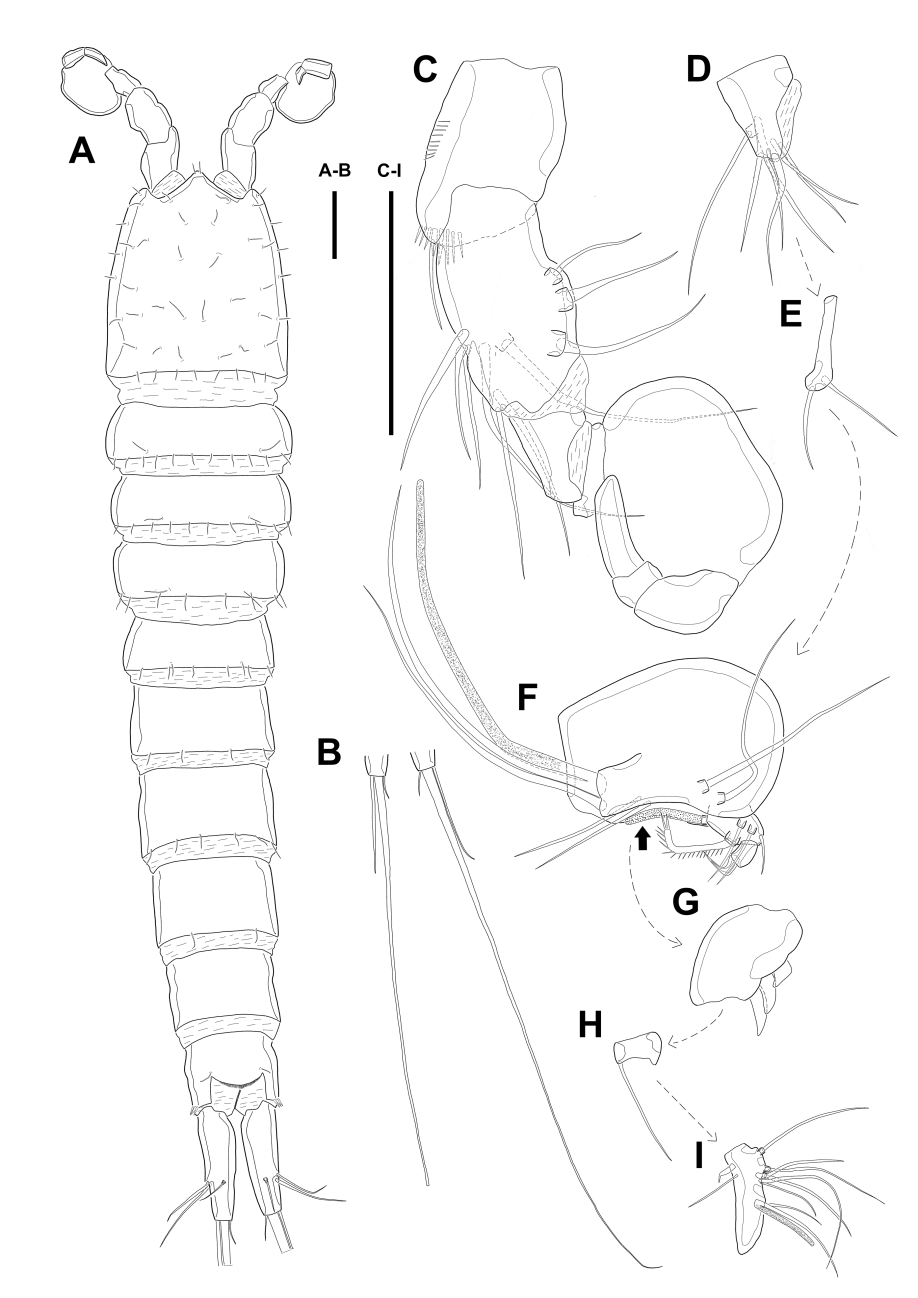
Line drawings of *Quinquelaophonte enormis* sp. nov. Male. (A–B) Paratype 8; (C–I) paratype 5). (A) Habitus, dorsal; (B) terminal part of caudal rami; (C) Antennule, dorsal; (D–I) third to eighth segment of antennule (arrow indicate a rod-like process in (F)), (D–F, I) dorsal, (F–G) ventral. Scale bars: (A–B), 100 µm; (C–I), 50 µm.

**Description of male.** Body (Figs. 5A–5B; 8A–8C) length, measured from anterior tip of rostrum to posterior margin of caudal rami, 691–774 µm (*n* = 7, mean = 729.43 µm). Urosome narrower than prosome. All body somite without hyaline frills (Figs. 5A; 8A) and entire surface, except for ventral surface, covered with tiny spiniform processes (Fig. 8A).

Prosome (Figs. 5A; 8A) four-segmented, comprising cephalothorax and three free pedigerous somites. Cephalothorax about as long as three succeeding prosomites combined. Rostrum small, fused to cephalothorax, and with two sensilla near anterior margin.

Urosome (Figs. 5B; 8A–8C) six-segmented, consisting of P5 bearing somite, P6 bearing somite, three free abdominal somites, anal somite and caudal rami, and gradually tapering posteriorly. Anal somite and caudal rami as in female (tube pore arrowed in Fig. 8C).

Several sensilla presented on the all of body somite except penultimate urosomite, as figured.

Antennule (Figs. 5C–5I; 8D–8E) eight-segmented, and subchirocer with geniculation between segments V and VI. Segment I (Fig. 5C) with one bare seta and armed with two rows of spinules on anterior surface dorsally and distal anterior corner ventrally, respectively. Terminal margin of dorsal surface much shorter than ventral side. Segment II (Fig. 5C) about twice as long as width and with three posterior setae arising from each setophore, two naked setae located on lateral margin, and three setae on the ventral surface, one of which arising from setophore. Segment III (Fig. 5D) sub-triangular shaped and with seven setae. Segment IV (Fig. 5E) shortest and with two setae. Segment V (Figs. 5F; 8D–8E) large, swollen, without processes on the dorsal surface, and with four setae, two of which located on anterior surface and other two of which arising from setophore on distal half, anteriorly. One rod-like process (arrowed in Fig. 5F) covered with dense bumps (indicated as dots in Fig. 5F; detailed in Fig. 8E) on anterior surface and two lobes presented on ventral surface and anterior margin, respectively. Ventral lobe with aesthetasc terminally and one naked seta. Anterior lobe two-segmented; proximal segment with two naked setae arising from each setophore on ventral surface, one bare seta on distal corner, and pectiniform process, as figured. Distal segment with one hook-like process terminally. Segment VI (Figs. 5G; 8D–8E) with three spinular processes. Segment VII (Figs. 5H; 8E) small and with one bare seta on distal corner. Segment VIII (Figs. 5I; 8E) sub-triangular shaped and with one spiniform process and seta on posterior margin distally. Anterior margin with one seta distally, five setae on distal half margin, two of which arising from each setophore, and acrothek consisting of blunt aesthetasc and two setae. All elements of terminal segment bare. Armature formula: 1-[1], 2-[10], 3-[7], 4-[2], 5-[7 + 1 pectiniform process + 1 hook-like process + rod-like process + (ae + 1)], 6-[3 spinous processes], 7-[1], 8-[7 + 1 spiniform process + acrothek].

Antenna to Maxilliped as in female.

P1 as in female.

**Figure 6:**
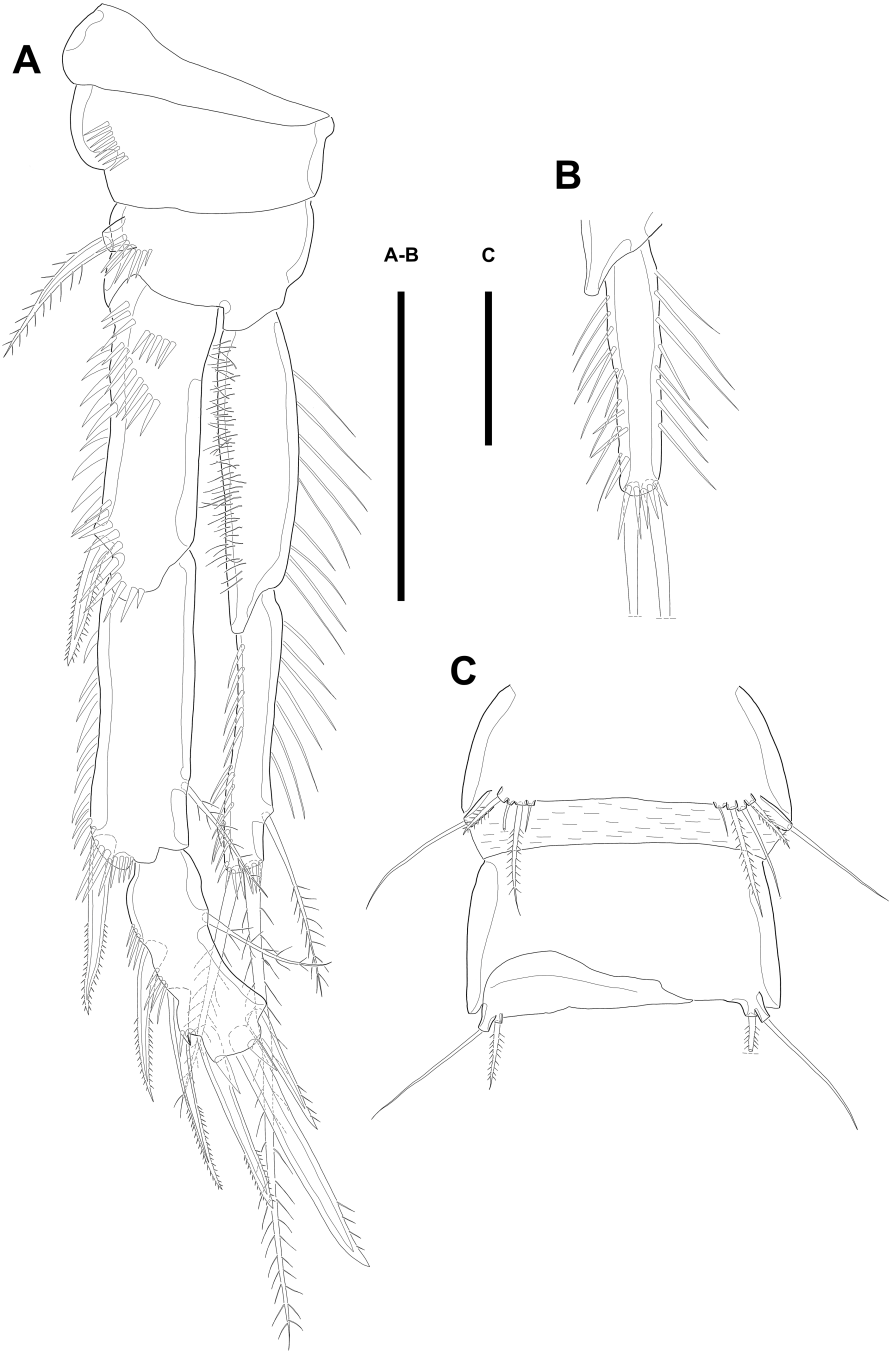
Line drawings of *Quinquelaophonte enormis* sp. nov. Male. (A, C: paratype 5 B paratype 6). (A) P2; (B) right distal segment of P2 enp; (C) urosomite bearing P5 and P6, ventral. Scale bars: 50 µm.

**Figure 7:**
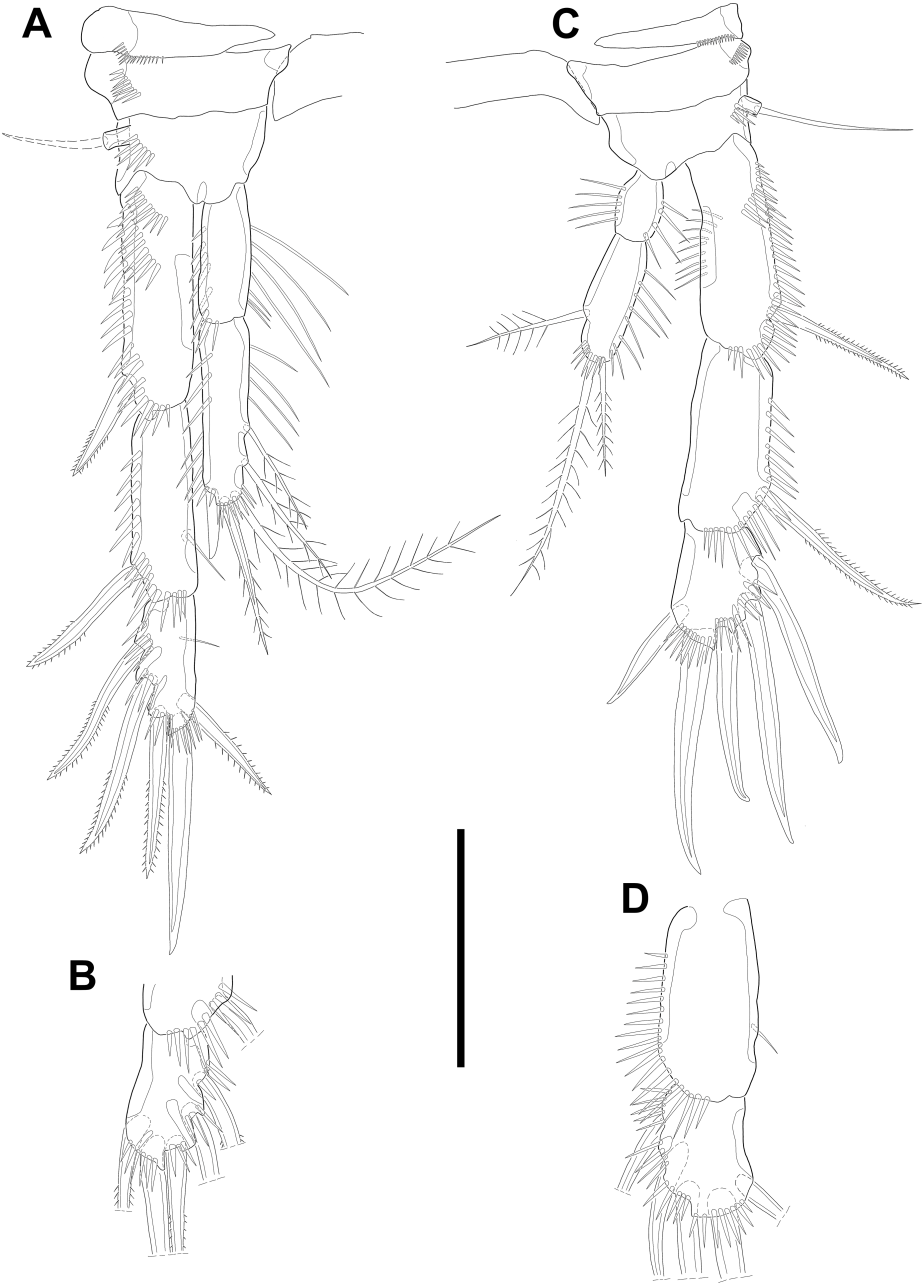
Line drawings of *Quinquelaophonte enormis* sp. nov. Male. (A, C) paratype 5; (B, D) paratype 6). (A) P3; (B) left distal segment of P3 exp; (C) P4; (D) right second and third segment of P4 exp. Scale bar: 50 µm.

Swimming legs P2–P4 (Figs. 6A–6B; 7) with wide intercoxal sclerite (illustration omitted in P2), three-segmented exopod, and two-segmented endopod. Praecoxa well developed, ornamented with one row of spinules near outer distal corner anteriorly, except for P2. Coxa armed with one row of spinules on outer margin, anteriorly. Exopod stouter than female.

P2 (Figs. 6A–6B). Basis with one bipinnate spine and ornamented with one row of spinules near base of spine. Exopod longer than endopod. Exp-1 as long as succeeding segment, approximately and armed with two rows of spinules from median surface to outer margin, anteriorly, and one row of spinules along outer margin. Bipinnate spine presented on outer distal corner of exp-1. Exp-2 with one plumose seta located on inner distal quarter and one bipinnate outer spine on terminal corner. Outer margin of exp-2 ornamented with one row of spinules. Exp-3 shortest and with one plumose inner seta, two unipinnate apical spines of which outer one about twice as long as inner, and three bipinnate outer spines. Two patches of spinules presented at bases of outer spines, and strong spinules existing on terminal margin. Endopod exceeding exp-2, slightly. Enp-1 marginally longer than enp-2 and ornamented with long setules along inner margin and mass of fine setules on outer surface, laterally. Tube pore presented on outer distal corner. Enp-2 with one plumose inner seta near terminal margin and two apical plumose setae, of which inner one longer than outer. Long setules and spinules presented as figured.

P3 (Figs. 7A–7B)*.* Outer margin of basis with one seta (omitted in illustration) arising from setophore of pedestal and ornamented with one row of spinules near setophore. Exopod longer than endopod. Exp-1 about 1.2 times longer than exp-2 and armed with two rows of spinules from median surface to outer margin, anteriorly and one row of spinules from anterior terminal margin to proximal of outer margin. Bipinnate spine presented on outer distal corner of exp-1. Exp-2 with one bare inner seta near distal half and bipinnate outer spine on terminal corner. Terminal and outer margin of exp-2 ornamented with spinules. Exp-3 shortest and with one tiny bare inner seta arising from posterior surface, two apical spines of which outer bare spine about 1.8 times as long as inner pinnate one, and three bipinnate outer spines. Several patches of spinules presented on base of each spine. Endopod reaching to half of exp-2. Enp-1 shorter than enp-2, ornamented with long setules along inner margin, several spinules on outer distal corner, anteriorly, and one row of spinules along outer margin. Enp-2 with two plumose inner setae and three terminal elements of which most outer seta modified to blunt spiniform process and among remaining two setae, inner one plumose and other one bipinnate. Setules and spinules presented as figured.

P4 (Figs. 7C–7D) with pedestal bearing one bare seta and spinular patch on outer margin of basis. Exopod about 2.7 times as long as endopod. Exp-1 longer than succeeding segment, with one bipinnate outer spine. Setules and spinules presented as figured. Exp-2 with one outer bipinnate spine and ornamented with spinules from terminal margin to proximal half of outer margin. Exp-3 shortest, about 1.3 times longer than width, and with three outer and two apical spines. Patches of spinules located near base of spines except for most outer and inner spines. Endopod slightly exceeding exp-1. Enp-1 ornamented with long setules along both of lateral margins. Enp-2 about twice as long as preceding segment and with one plumose inner seta on distal half and two apical setae, of which outer bipinnate one shorter than plumose inner. Spinular and setular row presented along anterior terminal margin and outer margin, respectively. Setal formulae of P2–P4 as in Table 1.

**Figure 8:**
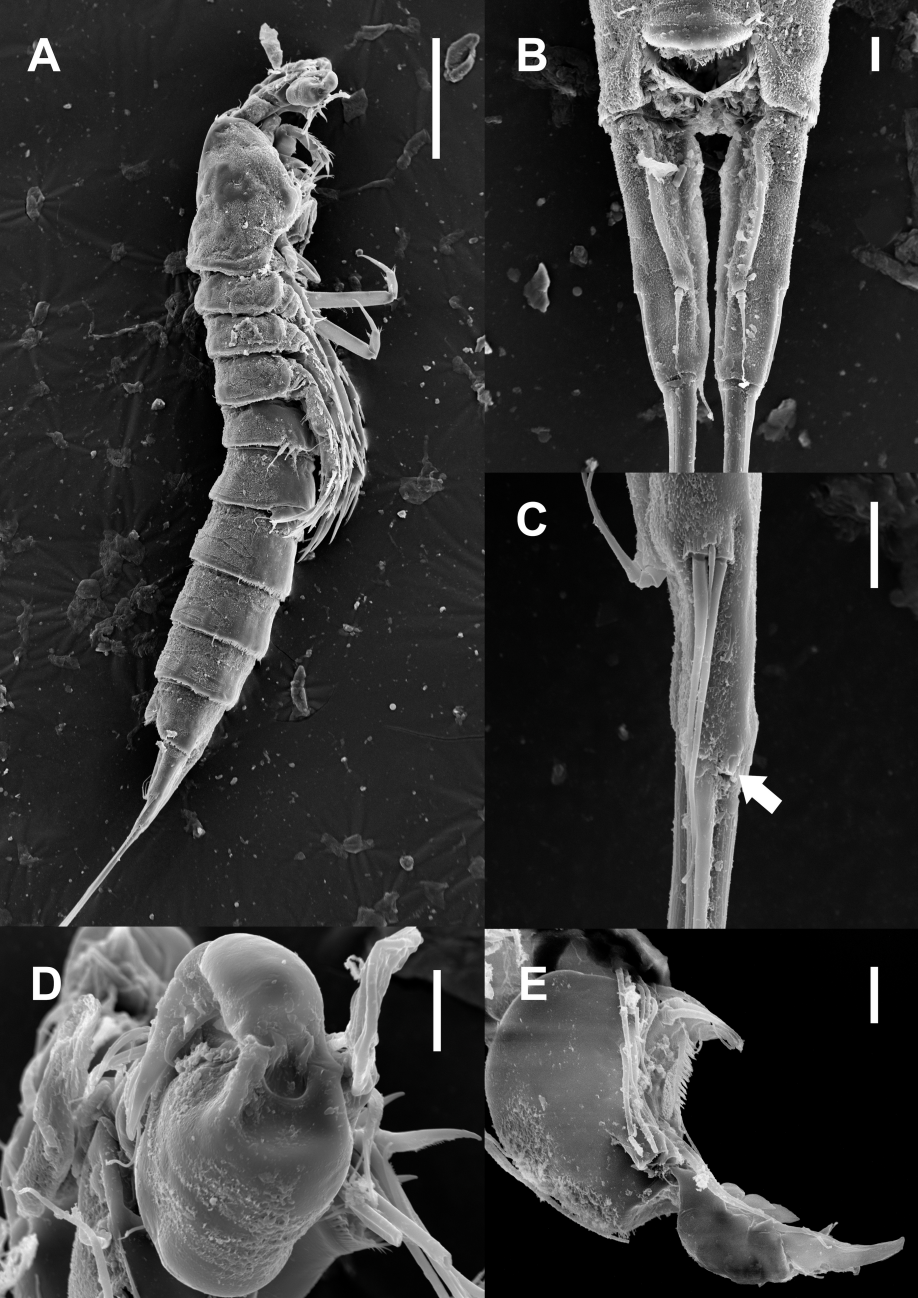
Scanning electron microscopy photographs of *Quinquelaophonte enormis* sp. nov. Male. (A–D) Paratype 13; (E) paratype 16. (A) Habitus, lateral; (B) caudal rami, dorsal; (C) right caudal ramus, lateral (arrow indicates a tube pore); (D) articulation of antennule, lateral; (E) fifth to eighth segment of antennule, ventral. Scale bars: (A), 100 µm; (B–D) 10 µm.

P5 (Fig. 6C) reduced and with five setae. Most outer seta arising from elongated pedestal and other four arising from each small protuberance of which most inner seta shortest.

P6 (Fig. 6C) asymmetrical and represented by small plate with two setae arising from each small protuberance. Inner bipinnate seta shorter than outer bare one. Right P6 presented in articulating genital lappet.

**Variability.** The morphological variation of *Quinquelaophonte enormis* **sp. nov.** appears to be complex. The individuals observed in this study differed in body size (see above) and the chaetotaxy of their swimming legs. To inspect the setal arrangement of the swimming legs of the new species, 13 specimens (five females and eight males) were examined. Intraspecific polymorphism was observed on P2–P4 within the population, between both sexes and even between the rami of each leg, asymmetrically (Figs. 4A–4D; 6A–6B; 7; detailed in Table 2).

**Table 2:** Armature formulae of segments in *Quinquelaophonte enormis***sp. nov.** with setal variation.

Specimens	Sex	Left/right ramus	P2 enp-3	P3 exp-3	P4 exp-2—3
Holotype	♀	Left	120	023	0.022
		Right	120	023	0.023
Paratype 1		L.	120	023	0.023
		R.	120	023	0.023
Paratype 2		L.	120	123	0.023
		R.	120	023	0.023
Paratype 3		L.	120	023	1.023
		R.	120	023	0.023
Paratype 4		L.	120	023	1.023
		R.	120	123	1.023
Paratype 5	♂	L.	120	123	0.023
		R.	120	123	0.023
Paratype 6		L.	120	023	0.023
		R.	020	023	1.023
Paratype 7		L.	120	123	0.023
		R.	120	123	0.023
Paratype 8		L.	120	023	0.023
		R.	020	023	1.023
Paratype 9		L.	120	023	0.023
		R.	120	023	0.023
Paratype 10		L.	120	123	0.023
		R.	120	023	0.023
Paratype 11		L.	120	123	damaged
		R.	120	023	1.023
Paratype 12		L.	120	123	0.023
		R.	120	123	0.023

**Etymology.** The specific name enormis refer to the setation of P2–P4 (Latin enōrmis, ‘irregular’).

**Molecular analysis.** The lengths of the sequence assembled for each gene were: (1) 18S: 1789 bp; (2) 28S: 742–777 bp (*n* = 2); (3) mtCOI: 664–676 bp (*n* = 6); (4) Cytb: 346–366 bp (*n* = 6). All new sequences were submitted to GenBank (18S: MT410708; 28S: MT420735 –MT420736; mtCOI: MT416598 –MT416603; Cytb: MT422734 –MT422739). A BLAST search of the GenBank database revealed that all obtained sequences were uncontaminated. In the blastn result, the data with the highest bit-score value for each locus were as follows: (1) 18S: *Quinquelaophonte aurantius* (score: 3053.49; accession number: MH444815; parent: Laophontidae); (2) 28S: *Pseudonychocamptus spinifer* (788.45; MF077863; Laophontidae); (3) mtCOI: *Quinquelaophonte aurantius* (647.79; MH444814; Laophontidae); (4) Cytb: *Danaus petilia* (139.24; KP007524; Insecta, Nymphalidae). In the mtCOI sequence, the *p*-distance was 0–0.003 between individuals of the new species and 0.188–0.191 between *Q. enormis* **sp. nov.** and *Q. aurantius* (Table S3). The *p*-distance of Cytb between individuals was 0–0.009 (Table S4).

**Figure 9:**
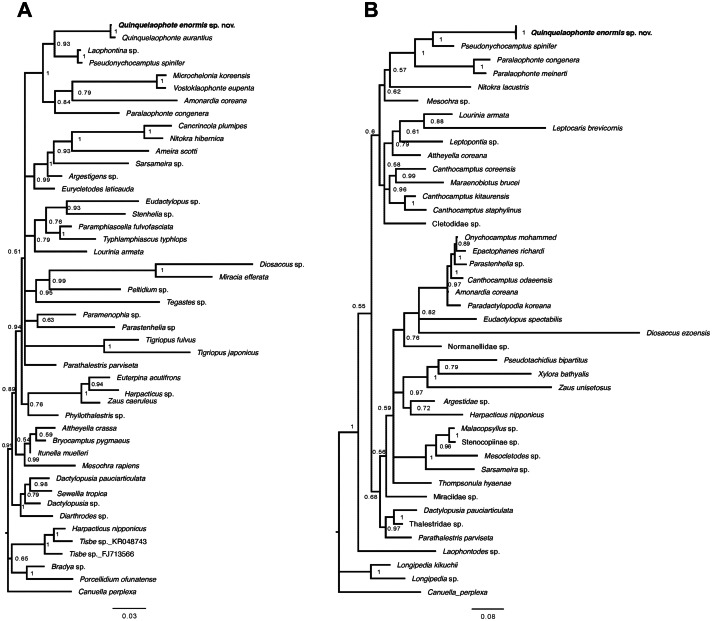
Bayesian trees of *Quinquelaophonte enormis* sp. nov. and other harpacticoids. (A) Based on partial 18S rRNA gene; (B) based on 28S rRNA gene. Numbers on each node are posterior probabilities. *Q. enormis* shown in bold. Scale bar: substitutions per site.

Two phylogenetic trees based on the partial sequences of 18S and 28S rRNA genes display that the relationships between some families of harpacticoid included in the data matrix remain unresolved (Fig. 9). The new species was grouped with the other laophontid genera/species in the two trees, although one miraciid species, *Amonardia coreana* (KT030261), was included in the tree generated using the partial of 18S rRNA gene (Fig. 9A). In the 18S tree, the clades for Laophontidae, including six genera, had strong statistical support (posterior probability, pp = 1) and were divided into two lineages (Fig. 9A). The genera *Laophontina*, *Pseudonychocamptus* and *Quinquelaophonte* formed a clade with high support (pp = 0.93), whereas the clade for the others was weakly supported (pp = 0.84) and included the *Amonardia* species mentioned above. The former lineage was divided into two clades including two *Quinquelaophonte* species, and the genera *Laophontina* and *Pseudonychocamptus*, respectively. The both clades were strongly supported (pp = 1). The tree based on 28S included three genera in the clade for Laophontidae (Fig. 9B). That clade was also divided into two groups, one with two *Paralaophonte* species, and the other with *Pseudonychocamptus spinifer* and the new species. Those two clades were also well supported (pp = 1).

## Discussion

When the genus *Quinquelaophonte* was erected by Wells, Hicks & Coull (1982) to encompass species included in the *quinquespinosa*-group, they added several characters to the diagnosis of the *quinquespinosa*-group. As a result, the diagnostic characters for *Quinquelaophonte* are: less than seven-segmented antennule of female; one-segmented exopod of antenna; single well-developed terminal caudal seta; considerable modification of male P2–P4 exopods; broad female P5 benp reaching end of exopod; female P5 with angular, not rounded exopod; unmodified endopod of male P2; male P5 represented by four or five setae on a small protuberance of segment margin. Because the new species described in this study has these all generic diagnostic characters, there is no doubt that it belongs in *Quinquelaophonte* and this result is also supported by molecular characters mentioned below.

*Quinquelaophonte enormis* **sp. nov.** is most similar to the Californian species, *Quinquelaophonte longifurcata* Lang, 1965. Those two species share the following characters: (1) three setae on the exopod of the antenna, length ratio over about 3.5 times of the caudal rami, and seven caudal setae (these characters were observed in most congeners, as detailed in Table 3); (2) the setal arrangement on the P4 exp-3 in both sexes (the formula, 023, was also reported in *Quinquelaophonte prolixasetae* Walker-Smith, 2004, but that species has five long setae and an long accessory seta on the P1 exp-2 and enp-2, respectively, as detailed in Table 1); (3) the absence of an inner seta on the male P4 exp-2 (in *Quinquelaophonte varians* Bjornberg, 2010, the inner seta is also absent, but that species has no inner seta on the male P3 exp-2 as its autapomorphic character, Table 1); (4) setation of the female P3 exp-3 (*Q. enormis* and *Q. longifurcata* are the only two species having the formula 023 for that segment, and both have similar aspect of variability of the inner seta). However, *Q. enormis* can be distinguished from *Q. longifurcata* by the following differences: (1) *Q. longifurcata*, but not *Q. enormis*, has an outer seta on the P4 enp-2 in both sexes (Table 1); (2) on the female P4 exp-2, an inner seta is present in the *Q. longifurcata* and lacking in the new species (Tables 1–2); (3) the new species has two setae on the syncoxa of the maxilliped, but *Q. longifurcata* has only one seta (Table 3). These differences indicate that *Q. enormis* is a distinct species. A comparison of morphological features among *Quinquelaophonte* species is given in Tables 1 and 3.

**Table 3:** Comparison of characters of *Quinquelaophonte* species, excluding *Q. brevicornis* (Scott T., 1894).

Species	References	No. of segments in ♀antennule	No. of setae					Length ratio of caudal rami
			Exp of antenna	Syncoxa of maxilliped	♀P5 exp: benp	♂P5	Caudal rami	
*Quinquelaophonte enormis***sp. nov.**	A	6	3	2	6:5	5	7	3.5
*Q. aestuarii*	B	6	3	2	6:5	5	6	4
*Q. aurantius*	C	6	3	2	6:5	5	7	3.5–3.8
*Q. bunakenensis*	D	6	3	1	6:5	4–5^a^	7	>2
*Q. candelabrum*	C; E	5	2	1	5:5	4	7	2
*Q. capillata*	F	–	–	–	–	5	–	–
	G	6	3	2	5:5	5	7	3–4
	H	6	3	1	6:5	5	7	3
*Q. koreana*	I	6	2	2	6:5	5	7	1
*Q. longifurcata*	J	6	3	1	6:5	5	7	4
*Q. parasigmoides*	K	6	3	–	6:5	5	7	2.5–3
*Q. prolixasetae*	L	6	3	2	6:5	5	7	3
*Q. quinquespinosa*	M	6	3–4^b^	1	6:5	5	6	2.5
	N	6	2	2	6:5	5	–	2
	H	6	3	2	6:5	–	7	2.3
*Q. varians*	O	6	3	1	6:5	5	6	4
*Q. wellsi*	P	6	3	2	6:5	5	6	2.7

**Notes.**

A this study B Sciberras, Bulnes & Cazzaniga, 2014 C Charry et al., 2019 D Mielke, 1997 E Wells, Hicks & Coull, 1982 F Wilson, 1932 G Coull, 1976; Coull, 1986 H Gómez & Morales-Serna, 2013 I Lee, 2003 J Lang, 1965 K Bozic, 1964; Bozic, 1969 L Walker-Smith, 2004 M Sewell, 1924 N Wells & McKenzie, 1973 O Bjornberg, 2010 P Hamond, 1973

a Mielke (1997) illustrated four setae in a figure but said an “outer lobe with a slender seta. Close to that lobe 2 slender and 2 strong setae are arranged”. in the text.

b Sewell (1924) figured three setae in a drawing but mentioned four in the description.

The first Korean species, *Q. koreana,* has a combination of morphological characters in both sexes also found in *Q. enormis*: (1) segmentation of the antennule in both sexes; (2) the number of setae on the syncoxa of the maxilliped; (3) the setal arrangement of P1–P2, endopod of P3, and P5; 4) the number of setae on the caudal rami. But that set of characters is shared by most of the congeners (Tables 1 and 3). On the other hand, *Q. koreana* and *Q. enormis* also have significant distinctions. *Q. koreana* has reduced features compared with the new species: two setae on the exopod antenna (three setae in the new species); caudal rami as long as width (about 3.5 times longer in the new species); and caudal seta V about half as long as the urosome (more than three fourths as long in the new species*;* Figs. 1C; 5B; 8B). The new species has other reduced features compared with *Q. koreana*: six or seven setae on P3 exp-3 in both sexes (eight setae in *Q. koreana*); no inner seta on P4 exp-3 in both sexes (inner seta present in *Q. koreana*); and no outer seta on P4 enp-2 of both sexes (outer seta exist in *Q. koreana*).

Interestingly, the reductions in the two Korean species appear, similarly, in the two New Zealand species, *Q. candelabrum* and *Q. aurantius*. Those two species share the setation of P1, P2, and P3 endopods, but those are also known in most of the congeneric species (Tables 1 and 3). Reductions in morphological features exist in both species. *Quinquelaophonte candelabrum* has a five-segmented antennule in the female (six-segmented in *Q. aurantius*); two setae on the exopod antenna (three setae in *Q. aurantius*); a seta on the syncoxa of the maxilliped (two setae in *Q. aurantius*); five baseoendopodal setae on the female P5 (six setae in *Q. aurantius*); four setae on the male P5 (five setae in *Q. aurantius*); and caudal rami twice as long as width (3.5–3.8 times longer in *Q. aurantius*). The reductions in *Q. aurantius* are: an inner seta on the P3 exp-3 in the female (two in *Q. candelabrum*); no inner seta on the male P3 exp-3 (one in *Q. candelabrum*); and no outer seta on the P4 enp-2 in both sexes (an outer seta on P4 enp-2 of *Q. candelabrum*). Among the five Pacific species, *Q. aurantius*, *Q. enormis*, and *Q. longifurcata* have the following common morphological traits: (1) fewer than two setae on the inner margin of P3 exp-3 (with two setae in the other two species); (2) no inner seta on P4 exp-3 in both sexes, except for the female of *Q. aurantius* (with at least one seta in the others); (3) caudal rami at least 3.5 times as long as width (at most twice in the others); (4) three exopodal setae on the antenna (two setae in the others). It seems that *Q. enormis* and *Q. aurantius* would be closer to each other, with *Q. longifurcata*, than the species described from each nearer location. However, it is not yet possible to determine which of these two groups is more derived because different reductions appear in both groups. Moreover, although a similar pattern of reduction is found in *Q. candelabrum* and *Q. koreana*, it is not yet able to conclude that they have experienced the same evolutionary history because it remains unknown whether their reduced characters are the result of divergence or convergence.

In the case of male P3 exp-3, all *Quinquelaophonte* species have at least one inner seta (Table 1). *Quinquelaophonte enormis* **sp. nov.** also has one inner seta; the presence of an inner seta on the left and right rami was observed in only three specimens. On the other hand, three other individuals had no inner seta on either ramus and the remaining two presented an inner seta on the left ramus only (Table 2). *Quinquelaophonte longifurcata* and *Q. aurantius*, grouped with the new species, have a seta on the inner margin of the segment, as mentioned above. But in the original description, only a single individual of the former species was investigated (Lang, 1965), and the existence of the inner seta in the latter was observed on only a single ramus in three of ten individuals (Charry et al., 2019). Furthermor, variability appears on the exopod of the male P4 in *Q. enormis* (Table 2). Among the eight observed individuals, three had an inner seta on the right ramus of P4 exp-2 (The examination was unavailable in one specimen due to damage on the left ramus. The other two had a seta on the right only). None of the specimens had polymorphism on exp-3. *Quinquelaophonte aurantius* was documented to have setal variation not only on the male P4 exp-2 but also on exp-3. In the original description, Charry et al. (2019) provided a setal formula for *Q. aurantius* with marks indicating the segment showing the variation. They also explained how many individuals had variable chaetotaxy in male P3 with the aspects of each specimen indicated in a table, but they did not provide equally detailed information of male P4. Although the results of their observations are omitted from the table, they do provide detailed accounts of exp-2–3 in the legend without clarifying which legs they mean. If their explanations indicate the setation of the male P4 exopod, they observed asymmetrical variation on P4 exp-2 in nine of ten individuals having an inner seta on only the left ramus and, the absence of an inner seta on exp-3 on both rami of nine specimens. In *Q. longifurcata*, no inner seta was described on the male P4 exp-2–3, but only a single male individual was investigated only. These situations cause ambiguity as to whether it has polymorphism or not, which is normal, and determination of character polarities within these Pacific species. More individuals of these species should be collected and examined in detail to resolve these uncertainties.

To investigate the genetic divergence between individuals of the new species, *p*-distance was measured. The results of those calculation for two mitochondrial genes show very small differences among six specimens (up to 0.3% in mtCOI and 0.9% in Cytb; Tables S3–S4). Among these paratype materials, two of Paratype 13 were, *in toto,* attached to a SEM stub without examination of the setal arrangement on the swimming legs unfortunately. The remaining materials, Paratypes 9–12, were observed and confirmed to have different arrangements (Table 2), but there is no genetic divergence between them for both loci (Tables S3–S4). This indicates that the *Quinquelaophonte* species described in this study is not a complex of species, despite the morphological variability.

A recent study by Charry et al. (2019) added 18S rRNA information for *Q. aurantius* to the data set used in Yeom et al. (2018) to analyze the evolutionary position of *Quinquelaophonte* within Laophontidae. As a result, they suggested that the genus *Quinquelaophonte* was closer to the genera *Laophontina* and *Pseudonychocamptus* than to *Microchelonia, Paralaophonte* and *Vostoklaophonte*. The results of our phylogenetic analysis using 18S rRNA gene in this study correspond well with the earlier studies and supported the placement of the new species in *Quinquelaophonte* (Fig. 9A). Another phylogenetic inference based on our 28S rRNA gene results also indicates that the genus *Quinquelaophonte* has a closer relationship with *Pseudonychocamptus* than *Paralaophonte* (Fig. 9B).

Because studies into the evolutionary relationships within family the Laophontidae have only recently begun using molecular methods, genetic information is still very limited despite its high diversity. Moreover, the small number of known DNA sequences is limited to particular loci, such as 18S rRNA, 28S rRNA and mtCOI. Furthermore, none of the mitochondrial genes generally used to delimit species and infer phylogenetic relationships within infrafamilial taxa such as 16S rRNA and Cytb, are available in GenBank. To increase understanding of the evolutionary relationships among *Quinquelaophonte* species and answer the morphological questions mentioned above, more molecular data from diverse loci should be accumulated.

## Conclusions

In summary, this is the second species of *Quinquelaophonte* species in Korea. *Quinquelaophonte enormis* **sp. nov.** shows resemblances to other Pacific species. We confirmed that the new species is a distinct species through morphological comparison, and we used molecular data to demonstrate that it is not a complex of species. To resolve ambiguities caused by complex morphological issue within the genus *Quinquelaophonte* and address issues caused by lack of genetic information about the family Laophontidae, more data need to be collected in further studies.

##  Supplemental Information

10.7717/peerj.10007/supp-1 Supplemental Information 1 Genetic information of *Quinquelaophonte enormis* sp. nov Accession numbers of Genbank, the length of sequences, type material information from which each sequence originated, name of each partial gene, and nucleotide sequence.

10.7717/peerj.10007/supp-2 Supplemental Information 2 Raw data for body measurements Body length of holotype to paratype 12, mean of length for each sex, collecting sites, where specimens are deposited, and deposit numbers for each specimen.

10.7717/peerj.10007/supp-3 Supplemental Information 3 GenBank accession numbers of 18S rDNA sequences used in this study

10.7717/peerj.10007/supp-4 Supplemental Information 4 GenBank accession numbers of 28S rDNA sequences used in this study

10.7717/peerj.10007/supp-5 Supplemental Information 5 Uncorrected pairwise distance for mtCOI among individuals of *Quinquelaophonte enormis* sp. nov. and *Q. aurantius*

10.7717/peerj.10007/supp-6 Supplemental Information 6 Uncorrected pairwise distance for Cytb among individuals of *Quinquelaophonte enormis* sp. nov

## Additional Information and Declarations

### DNA Deposition

The following information was supplied regarding the deposition of DNA sequences: The partial sequences of 18S ribosomal RNA gene are available at GenBank: MT410708, the partial sequences of 28S ribosomal RNA gene are available at GenBank: MT420735 to MT420736, the partial sequences of mitochondrial cytochrome c oxidase subunit I are available at GenBank: MT416598 to MT416603, and the partial sequences of cytochrome b are available at GenBank: MT422734 to MT422739. The sequencing data are available in the Supplemental Files.

### Data Availability

The following information was supplied regarding data availability: The genetic information used in this study are available in File S1. The body length for each specimen are available in File S2. All materials prepared in this study were deposited in the National Institute of Biological Resources (Incheon, Korea). All type materials were given a deposit number from NIBRIV0000865933 to 0000865949. The meaning of abbreviation is: NIBR, National Institute of Biological Resources; IV, invertebrate. Holotype: 1♀ on a slide (NIBRIV0000865933) Paratypes: Paratype 1, 1♀ dissected on four slides (NIBRIV0000865934); Paratype 2, 1♀ dissected on seven slides (NIBRIV0000865935); Paratype 3, 1♀ dissected on 10 slides (NIBRIV0000865936); Paratype 4, 1♀ dissected on nine slides (NIBRIV0000865937); Paratype 5, 1♂ dissected on eight slides (NIBRIV0000865938); Paratype 6, 1♂ dissected on six slides (NIBRIV0000865939); Paratypes 7–12, 6♂♂ on each slide (NIBRIV0000865940–0000865945); Paratype 13, 2♂♂, in toto, on a SEM stub (NIBRIV0000865946); Paratypes 14–16, 1♀ 2♂♂ on each SEM stubs (NIBRIV0000865947–0000865949).

### New Species Registration

The following information was supplied regarding the registration of a newly described species: Publication LSID: urn:lsid:zoobank.org:pub:D3FBC69D-D672-459C-A7F3-4ADBC92C5615; *Quinquelaophonte enormis* **sp. nov.** LSID: urn:lsid:zoobank.org:act:7094B4E4-DE75-4C9B-8746-F02EB077B579.
