# Supplementary material for: Quinquelaophonte enormis sp. nov., a new interstitial copepod (Harpacticoida: Laophontidae) from Korea
Source: PeerJ. 2020 Sep 22;8:e10007. doi: 10.7717/peerj.10007 (PMC7518157; doi:10.7717/peerj.10007)
Supplement: Supplemental Information 3 [file peerj-08-10007-s003.docx]

Table S1. GenBank accession numbers of 18S rDNA sequences used in this study.

| Family | Species | Accession# | Reference |
| --- | --- | --- | --- |
| Ameiridae | *Ameira scotti* | EU380303 | Huys, Mackenzie-Dodds & Llewellyn-Hughes, 2009 |
|  | *Cancrincola plumipes* | L81938 | Spears & Abele, 1997 |
|  | *Nitokra hibernica* | EU380305 | Huys, Mackenzie-Dodds & Llewellyn-Hughes, 2009 |
|  | *Sarsameira* sp. | EU380304 | Huys, Mackenzie-Dodds & Llewellyn-Hughes, 2009 |
| Argestidae | *Argestigens* sp. | EU380306 | Huys, Mackenzie-Dodds & Llewellyn-Hughes, 2009 |
|  | *Eurycletodes laticauda* | EU380310 | Huys, Mackenzie-Dodds & Llewellyn-Hughes, 2009 |
| Canthocamptidae | *Attheyella crassa* | EU380307 | Huys, Mackenzie-Dodds & Llewellyn-Hughes, 2009 |
|  | *Bryocamptus pygmaeus* | AY627015 | Huys et al., 2006 |
|  | *Itunella muelleri* | EU380309 | Huys, Mackenzie-Dodds & Llewellyn-Hughes, 2009 |
|  | *Mesochra rapiens* | EU380308 | Huys, Mackenzie-Dodds & Llewellyn-Hughes, 2009 |
| Canuellidae | *Canuella perplexa* | EU370432 | von Reumont et al., 2009 |
| Dactylopusiidae | *Dactylopusia pauciarticulata* | KR048735 | Baek and Hwang, Unpublished |
|  | *Dactylopusia* sp. | EU380295 | Huys, Mackenzie-Dodds & Llewellyn-Hughes, 2009 |
|  | *Diarthrodes* sp. | EU380296 | Huys, Mackenzie-Dodds & Llewellyn-Hughes, 2009 |
|  | *Sewellia tropica* | EU380299 | Huys, Mackenzie-Dodds & Llewellyn-Hughes, 2009 |
| Ectinosomatidae | *Bradya* sp. | AY627016 | Huys et al., 2006 |
| Harpacticidae | *Harpacticus nipponicus* | KR048736 | Baek and Hwang, Unpublished |
|  | *Harpacticus* sp. | EU380285 | Huys, Mackenzie-Dodds & Llewellyn-Hughes, 2009 |
|  | *Tigriopus fulvus* | EU370430 | von Reumont et al., 2009 |
|  | *Tigriopus japonicus* | EU054307 | Ki et al., 2009 |
|  | *Zaus caeruleus* | EU380284 | Huys, Mackenzie-Dodds & Llewellyn-Hughes, 2009 |
| Laophontidae | *Laophontina* sp. | MF077713 | Khodami et al., 2017 |
|  | *Microchelonia koreensis* | MG012752 | Yeom et al., 2018 |
|  | *Paralaophonte congenera* | KR048738 | Baek and Hwang, Unpublished |
|  | *Pseudonychocamptus spinifer* | MF077714 | Khodami et al., 2017 |
|  | *Quinquelaophonte aurantius* | MH444815 | Charry et al., 2019 |
|  | *Quinquelaophonte enormis* | MT410708 | this study |
|  | *Vostoklaophonte eupenta* | MG012753 | Yeom et al., 2018 |
| Louriniidae | *Lourinia armata* | KR048739 | Baek and Hwang, Unpublished |
| Miraciidae | *Amonardia coreana* | KT030261 | Baek and Hwang, Unpublished |
|  | *Diosaccus* sp. | EU380290 | Huys, Mackenzie-Dodds & Llewellyn-Hughes, 2009 |
|  | *Miracia efferata* | EU380294 | Huys, Mackenzie-Dodds & Llewellyn-Hughes, 2009 |
|  | *Paramphiascella fulvofasciata* | EU380293 | Huys, Mackenzie-Dodds & Llewellyn-Hughes, 2009 |
|  | *Stenhelia* sp. | EU380291 | Huys, Mackenzie-Dodds & Llewellyn-Hughes, 2009 |
|  | *Typhlamphiascus typhlops* | EU380292 | Huys, Mackenzie-Dodds & Llewellyn-Hughes, 2009 |
| Parastenheliidae | *Parastenhelia* sp. | EU380302 | Huys, Mackenzie-Dodds & Llewellyn-Hughes, 2009 |
| Peltidiidae | *Peltidium* sp. | EU380288 | Huys, Mackenzie-Dodds & Llewellyn-Hughes, 2009 |
| Porcellidiidae | *Porcellidium ofunatense* | KR048741 | Baek and Hwang, Unpublished |
| Tachidiidae | *Euterpina acutifrons* | GU969212 | Wang & Sun, Unpublished |
| Tegastidae | *Tegastes* sp. | EU380287 | Huys, Mackenzie-Dodds & Llewellyn-Hughes, 2009 |
| Thalestridae | *Eudactylopus* sp. | EU380301 | Huys, Mackenzie-Dodds & Llewellyn-Hughes, 2009 |
|  | *Paramenophia* sp. | EU380300 | Huys, Mackenzie-Dodds & Llewellyn-Hughes, 2009 |
|  | *Parathalestris parviseta* | KR048742 | Baek and Hwang, Unpublished |
|  | *Phyllothalestris* sp. | EU380298 | Huys, Mackenzie-Dodds & Llewellyn-Hughes, 2009 |
| Tisbidae | *Tisbe* sp. | FJ713566 | Chullasorn et al., 2011 |
|  | *Tisbe* sp. | KR048743 | Baek and Hwang, Unpublished |

Charry MP, Wells JBJ, Keesing V, Smith KF, Stringer TJ, Tremblay LA. 2019. *Quinquelaophonte* *aurantius* sp. nov., a new harpacticoid species (Copepoda: Harpacticoida: Laophontidae: *Quinquelaophonte*) from New Zealand. *New Zealand Journal of Zoology* 46(4): 301-320.

Chullasorn S, Dahms H, Lee K, Ki J, Schizas N, Kangtia P, Park HG, Lee J. 2011. Description of Tisbe alaskensis sp. nov. (Crustacea: Copepoda) Combining Structural and Molecular Traits. *Zoological Studies* 50(1): 103-117.

Huys R, Mackenzie-Dodds J, Llewellyn-Hughes J. 2009. Cancrincolidae (Copepoda, Harpacticoida) associated with land crabs: A semiterrestrial leaf of the ameirid tree. *Molecular Phylogenetics and* *Evolution* 51: 143-156.

Huys R, Llewellyn-Hughes J, Olson PD, Nagasawa K. 2006. Small subunit rDNA and Bayesian inference reveal *Pectenophilus ornatus* (Copepoda *incertae sedis*) as highly transformed Mytilicolidae, and support assignment of Chondracanthidae and Xarifiidae to Lichomolgoidea (Cyclopoida). *Biological Journal of the Linnean Society* 87(3): 403-425.

Khodami S, McArthur JV, Blanco-Bercial L, Marinez Arbizu P. 2017. Molecular Phylogeny and Revision of Copepod Orders (Crustacea: Copepoda). *Scientific Reports* 7: 9164.

Ki J, Lee K, Park HG, Chullasorn s, Dahms H, Lee J. 2009. Phylogeography of the copepod *Tigriopus japonicus* along the Northwest Pacific rim. *Journal of Plankton Research* 31(2): 209-221.

Spears T, Abele LG. 1997. Crustacean phylogeny inferred from 18S rDNA. In: Fortey RA, Thomas RH, ed. *Arthropoda Relationships*. London: Chapman & Hall, 169-187.

von Reumont BM, Meusemann K, Szucsich NU, Dell’Ampio E, Gowri-Shankar V, Bartel D, Simon S, Letsch HO, Stocsits RR, Luan Y, Wägele JW, Pass G, Hadrys H, Misof B. 2009. Can comprehensive background knowledge be incorporated into substitution models to improve phylogenetic analyses? A case study on major arthropod relationships. *BMC Evolutionary Biology* 9: 119.

Yeom J, Nikitin MA, Ivanenko VN, Lee W. 2018. A new minute ectosymbiotic harpacticoid copepod living on the sea cucumber *Eupentacta fraudatrix* in the East/Japan Sea. *Peer J* 6:e4979.
