# Supplementary material for: Quinquelaophonte enormis sp. nov., a new interstitial copepod (Harpacticoida: Laophontidae) from Korea
Source: PeerJ. 2020 Sep 22;8:e10007. doi: 10.7717/peerj.10007 (PMC7518157; doi:10.7717/peerj.10007)
Supplement: Supplemental Information 4 [file peerj-08-10007-s004.docx]

Table S2. GenBank accession numbers of 28S rDNA sequences used in this study.

| Family | Species | Accession# | Reference |
| --- | --- | --- | --- |
| Ameiridae | *Malacopsyllus* sp. | MF077803 | Khodami et al., 2017 |
|  | *Nitokra lacustris* | KR048883 | Baek and Hwang, Unpublished |
|  | *Sarsameira* sp. | MF077836 | Khodami et al., 2017 |
|  | Stenocopiinae sp. | MF077794 | Khodami et al., 2017 |
| Ancorabolidae | *Laophontodes* sp. | MF077795 | Khodami et al., 2017 |
| Argestidae | Argestidae sp. | MF077831 | Khodami et al., 2017 |
|  | *Mesocletodes* sp. | MF077844 | Khodami et al., 2017 |
| Canthocamptidae | *Attheyella coreana* | KR048885 | Baek and Hwang, Unpublished |
|  | *Canthocamptus coreensis* | KR048886 | Baek and Hwang, Unpublished |
|  | *Canthocamptus kitaurensis* | KR048887 | Baek and Hwang, Unpublished |
|  | *Canthocamptus odaeensis* | KR048888 | Baek and Hwang, Unpublished |
|  | *Canthocamptus staphylinus* | MF077853 | Khodami et al., 2017 |
|  | *Epactophanes richardi* | KR048889 | Baek and Hwang, Unpublished |
|  | *Maraenobiotus brucei* | KR048890 | Baek and Hwang, Unpublished |
|  | *Mesochra* sp. | MF077802 | Khodami et al., 2017 |
| Canuellidae | *Canuella perplexa* | MF109111 | Khodami et al., 2017 |
| Cletodidae | Cletodidae sp. | MF077804 | Khodami et al., 2017 |
| Dactylopusiidae | *Dactylopusia pauciarticulata* | KR048872 | Baek and Hwang, Unpublished |
|  | *Paradactylopodia koreana* | KR048891 | Baek and Hwang, Unpublished |
| Darcythompsoniidae | *Leptocaris brevicornis* | KR048871 | Baek and Hwang, Unpublished |
| Harpacticidae | *Harpacticus nipponicus* | KR048873 | Baek and Hwang, Unpublished |
|  | *Zaus unisetosus* | KR048874 | Baek and Hwang, Unpublished |
| Laophontidae | *Onychocamptus mohammed* | KR048892 | Baek and Hwang, Unpublished |
|  | *Paralaophonte congenera* | KR048875 | Baek and Hwang, Unpublished |
|  | *Paralaophonte meinerti* | KR048898 | Baek and Hwang, Unpublished |
|  | *Pseudonychocamptus spinifer* | MF077863 | Khodami et al., 2017 |
|  | *Quinquelaophonte enormis* | MT420735-420736 | this study |
| Leptopontiidae | Leptopontia sp. | MF077852 | Khodami et al., 2017 |
| Longipediidae | *Longipedia kikuchii* | KR048876 | Baek and Hwang, Unpublished |
|  | *Longipedia* sp. | MF109112 | Khodami et al., 2017 |
| Louriniidae | *Lourinia armata* | KR048877 | Baek and Hwang, Unpublished |
| Miraciidae | *Amonardia coreana* | KR048894 | Baek and Hwang, Unpublished |
|  | *Diosaccus ezoensis* | KR048878 | Baek and Hwang, Unpublished |
|  | Miraciidae sp. | MF077845 | Khodami et al., 2017 |
| Normanellidae | Normanellidae sp. | MF077857 | Khodami et al., 2017 |
| Parastenheliidae | *Parastenhelia* sp. | KR048895 | Baek and Hwang, Unpublished |
| Pseudotachidiidae | *Pseudotachidius bipartitus* | MF077833 | Khodami et al., 2017 |
|  | *Xylora bathyalis* | MF077806 | Khodami et al., 2017 |
| Thalestridae | *Eudactylopus spectabilis* | KR048897 | Baek and Hwang, Unpublished |
|  | *Parathalestris parviseta* | KR048880 | Baek and Hwang, Unpublished |
|  | Thalestridae sp. | MF077796 | Khodami et al., 2017 |
| Thompsonulidae | *Thompsonula hyaenae* | MF077864 | Khodami et al., 2017 |

Khodami S, McArthur JV, Blanco-Bercial L, Marinez Arbizu P. 2017. Molecular Phylogeny and Revision of Copepod Orders (Crustacea: Copepoda). *Scientific Reports* 7: 9164.
