# Supplementary material for: Quinquelaophonte enormis sp. nov., a new interstitial copepod (Harpacticoida: Laophontidae) from Korea
Source: PeerJ. 2020 Sep 22;8:e10007. doi: 10.7717/peerj.10007 (PMC7518157; doi:10.7717/peerj.10007)
Supplement: Supplemental Information 5 [file peerj-08-10007-s005.docx]

|  | 1 | 2 | 3 | 4 | 5 | 6 |
| --- | --- | --- | --- | --- | --- | --- |
| 1. Paratype 9  (accession number: MT416598) |  |  |  |  |  |  |
| 2. Paratype 10 (MT416599) | 0 |  |  |  |  |  |
| 3. Paratype 11 (MT416600) | 0 | 0 |  |  |  |  |
| 4. Paratype 12 (MT416601) | 0 | 0 | 0 |  |  |  |
| 5. Paratype 13_1 (MT416602) | 0.001 | 0.001 | 0.001 | 0.001 |  |  |
| 6. Paratype 16 (MT416603) | 0.003 | 0.003 | 0.003 | 0.003 | 0.002 |  |
| 7. *Q. aurantius* (MH444814) | 0.188 | 0.189 | 0.188 | 0.188 | 0.19 | 0.191 |

Table S3. Uncorrected pairwise distance for mtCOI among individuals of *Quinquelaophonte enormis* **sp. nov.** and *Q. aurantius*.
