# Supplementary material for: Quinquelaophonte enormis sp. nov., a new interstitial copepod (Harpacticoida: Laophontidae) from Korea
Source: PeerJ. 2020 Sep 22;8:e10007. doi: 10.7717/peerj.10007 (PMC7518157; doi:10.7717/peerj.10007)
Supplement: Supplemental Information 6 [file peerj-08-10007-s006.docx]

Table S4. Uncorrected pairwise distance for Cytb among individuals of *Quinquelaophonte enormis* **sp. nov.**

|  | 1 | 2 | 3 | 4 | 5 |
| --- | --- | --- | --- | --- | --- |
| 1. Paratype 9  (accession number: MT422734) |  |  |  |  |  |
| 2. Paratype 10 (MT422735) | 0 |  |  |  |  |
| 3. Paratype 11 (MT422736) | 0 | 0 |  |  |  |
| 4. Paratype 12 (MT422737) | 0 | 0 | 0 |  |  |
| 5. Paratype 13_1 (MT422738) | 0.003 | 0.003 | 0.003 | 0.003 |  |
| 6. Paratype 13_2 (MT422739) | 0.009 | 0.009 | 0.009 | 0.009 | 0.006 |
